# How to Identify Roast Defects in Coffee Beans Based on the Volatile Compound Profile

**DOI:** 10.3390/molecules27238530

**Published:** 2022-12-03

**Authors:** Robert Rusinek, Bohdan Dobrzański, Anna Oniszczuk, Marzena Gawrysiak-Witulska, Aleksander Siger, Hamed Karami, Aneta A. Ptaszyńska, Aleksandra Żytek, Krzysztof Kapela, Marek Gancarz

**Affiliations:** 1 Institute of Agrophysics Polish Academy of Sciences, Doświadczalna 4, 20-290 Lublin, Poland; 2 Pomology, Nursery and Enology Department, University of Life Sciences in Lublin, Głęboka 28, 20-400 Lublin, Poland; 3 Department of Inorganic Chemistry, Medical University of Lublin, Chodźki 4a, 20-093 Lublin, Poland; 4 Department of Dairy and Process Engineering, Poznan University of Life Sciences, Wojska Polskiego 28, 60-637 Poznan, Poland; 5 Department of Food Biochemistry and Analysis, Poznan University of Life Sciences, Wojska Polskiego 28, 60-637 Poznan, Poland; 6 Department of Biosystems Engineering, University of Mohaghegh Ardabili, Ardabil 56199-11367, Iran; 7 Department of Immunobiology, Institute of Biological Sciences, Faculty of Biology and Biotechnology, Maria Curie-Skłodowska University, Akademicka 19 Str., 20-033 Lublin, Poland; 8 Faculty of Agrobioengineering and Animal Husbandry, University of Natural Sciences and Humanities in Siedlce, ul. Prusa 14, 08-110 Siedlce, Poland; 9 Faculty of Production and Power Engineering, University of Agriculture in Krakow, Balicka 116B, 30-149 Krakow, Poland

**Keywords:** electronic nose, GC–MS, roast defects of coffee beans, Coffee Arabica

## Abstract

The aim of this study was to detect and identify the volatile compounds in coffee that was obtained in defect roast processes versus standard roasting and to determine the type and strength of the correlations between the roast defects and the volatile compound profile in roasted coffee beans. In order to achieve this goal, the process of coffee bean roasting was set to produce an underdeveloped coffee defect, an overdeveloped coffee defect, and defectless coffee. The “Typica” variety of Arabica coffee beans was used in this study. The study material originated from a plantation that is located at an altitude of 1400–2000 m a.s.l. in Huehuetenango Department, Guatemala. The analyses were carried out with the use of gas chromatography/mass spectrometry (GC–MS) and an electronic nose. This study revealed a correlation between the identified groups of volatile compounds and the following coffee roasting parameters: the time to the first crack, the drying time, and the mean temperatures of the coffee beans and the heating air. The electronic nose helped to identify the roast defects.

## 1. Introduction

Coffee is one of the most popular beverages in the world. Coffee consumption is steadily increasing, and its average monthly price, which was estimated at USD 1.9517 per pound in November 2021, is also rising constantly [1]. The leading coffee producers include Brazil (3,804,000 t), Vietnam (1,740,000 t), Indonesia (717,000 t), and Colombia (858,000 t). In turn, the USA (1,618,900 t), the EU (2,415,000 t), Brazil (1,344,000 t), and Japan (443,200 t) are the largest coffee consumers [1]. 

Coffee has a very distinctive aroma due to its content of volatile compounds. The concentration of VOCs (volatile organic compounds) may change depending on the temperature that is used during the coffee roasting process [2,3]. The size of the ground particles and the coffee brewing methods are equally important. The chemical reactions and the changes in the content of the compounds exert a significant effect on the flavor, the aroma, and the color of the beverage [4]. Similarly, the type of coffee, the country of origin, and the growing, harvesting, and storage conditions have a considerable impact on the profile of the volatile compounds, flavor, and aroma [5,6]. For example, Colombian coffees contain a higher concentration of sulfur compounds, e.g., dimethyl disulfide, which is responsible for the cabbage-like aroma, whereas coffees from Ethiopia have higher concentrations of monoterpenes (citrus aroma) [7]. 

Coffee has a rich composition that affects its flavor and aroma. It contains carbohydrates, lipids, vitamins, nitrogen compounds, minerals, alkaloids, and phenolic compounds. The consumption of coffee is associated with many positive effects, which are exerted by bioactive substances such as caffeine, chlorogenic acid, diterpenes, and trigonelline [8]. Coffee is the main dietary source of chlorogenic acid; hence, it can serve as an effective antioxidant. Coffee consumption can reduce the risk of DNA damage-related malignancies, i.e., colon or oral cavity cancers. Moreover, it inhibits the development of some diseases, e.g., diabetes and liver diseases, protects against Parkinson’s disease, promotes wound healing, reduces inflammation, and inhibits the oxidation of LDL cholesterol [9,10]. 

The most numerous groups of volatile substances are furans and pyrazines, the content of which depends on the degree of roasting. The caffeine content changes together with the increase in the coffee roasting degree, i.e., the higher the roasting degree, the lower the content of this alkaloid. The amount of caffeine in roasted coffee might also slightly increase compared to the green form. These minimal changes in caffeine can be caused due to sublimation (overheating) [11,12]. The single compounds that are contained in roasted coffee do not have the typical flavor and aroma, but their mixtures generate a unique aroma [13,14]. The chemical reactions taking place during coffee bean roasting through which important volatile substances are released include Maillard reactions (enzymatic browning) and the degradation of phenolic acids and carotenoids [15].

The roasting process may sometimes be burdened by errors that have a direct impact on the quality of the coffee. Roast defects affecting the aroma and the flavor of the coffee infusions may arise during the production process or may be caused by roasting old coffee beans or if the material is stored in inappropriate conditions. In turn, differences in the roasting time and the temperature affect the rate of moisture loss, the internal temperature, and the chemical composition of coffee beans [16]. This may lead to frequent problems in coffee bean roasting, such as the partial carbonization of coffee beans, overdevelopment, or uneven temperature distribution in coffee beans. Furthermore, conventional roasters are considered to be unsanitary due to coffee bean roasting at excessively high temperatures and the deposition of oil, which makes the cleaning and the operation of the roaster difficult [17]. 

Many factors, i.e., the species, the variety, and the cultivation conditions (climate, season, location of plantations, height above sea level, impact of oceans, substrate and soil, humidity and rainfall, presence of other plant), modify the quality and the properties of coffee beans (their size, shape, hardness, and density) [18]. This requires the selection of the initial parameters of the roaster and the bean roasting process each time. In an attempt to obtain the desired roasting degree, also depending on the consumer preferences and the coffee brewing methods, the roasting process can easily become defective. The defects can be recognized by an experienced roaster, but the determination thereof based on the chemical properties and the profile of the aromatic compounds can help to recognize often invisible defects that deteriorate the flavor of the beverage. 

There are several defects that may arise during coffee roasting that may yield baked, underdeveloped, overdeveloped, and scorched coffee [16,19,20]. The baked coffee defect occurs when the coffee beans are heated for too long with hot air at an insufficient temperature without reaching the first crack. This defect is referred to as stalling the roast. It is invisible, but the roasted beans have a characteristic flat flavor with little sweetness, which is often described as a bread-like or papery flavor. Underdeveloped beans are usually grassy and devoid of the caramelized sugars that are present during the roasting process. This defect usually occurs in the light roast process, which is carried out at a slightly insufficient temperature. It sometimes occurs in the production of light roast coffee beans. The overdeveloped coffee defect is the opposite of underdeveloped coffee. It is a result of treatment with a slightly excessive temperature, as in the case of the Vienna or French roasting styles. There is a slight difference between a darker roast (Vienna, French) and the overdeveloped coffee. In both cases, the beans will look dark and greasy, sometimes even nearly black, but the flavor of the coffee that is brewed from overdeveloped beans will be burnt and bitter with smoky and coal notes [21]. Scorched coffee is produced when the charge temperature, i.e., the starting temperature, is too high and the speed of the drum is too slow. In such a case, dark, burnt stains appear on the coffee bean surface, and the coffee may taste oily, smoky, and reminiscent of roasted poultry [21].

The aim of this study was to determine and identify the volatile compounds that are generated in the process of defective coffee roasting. In order to achieve this goal, coffee beans were roasted in conditions causing the most common roast defects, i.e., insufficient and excessive temperatures. Thus, coffee underdevelopment and overdevelopment defects were achieved. Additionally, the beans from the same batch were roasted in appropriate conditions, yielding a comparative material, i.e., coffee that was roasted according to common standards. This study was conducted with the use of mass spectrometry with gas chromatography (GC–MS) and an electronic nose. The information obtained in this way will allow the indication of the major volatile compounds as a marker of a given roasting defect.

## 2. Results

### 2.1. Analysis of Volatile Compounds in Green Coffee

The analysis of the volatile compounds that were contained in the green coffee beans was carried out separately from the analysis of the roasted coffee due to the different nature of the material, which was not subjected to the roasting process and did not contain volatile compounds that are typical of roasted coffee beans [15,22]. Lower numbers of volatile compounds were determined in the green coffee beans in comparison with the roasted beans due to the lack of compounds that were generated during the roasting process, which determined the final aroma of the coffee. This is consistent with the results of similar studies [23,24]. Green coffee beans only have a basic VOC composition compared to roasted beans. While over 1000 volatile compounds are typically detected in roasted coffee, only approximately 200 VOCs are identified in green beans. These compounds, and other aroma molecules, are mainly generated during the roasting process from the non-volatile precursors that are present in green coffee beans, e.g., polysaccharides, lipids, proteins, and free amino acids [25].

Table 1 presents 18 volatile compounds that were identified in the green coffee beans. The volatile compounds were classified into groups of chemical compounds, which were present in the following amounts: alcohols—12.5%, acids—5%, ketones—1.4%, azines—4.3%, esters—47.1%, amines—4.6%, terpenes—11.4%, hydrocarbons—11.8%, others—1.9%, and furans, aldehydes, and pyranes—0%. The group of volatile compounds that were contained in the green coffee was represented predominantly by esters, while unidentified substances accounted for the lowest percentage. No furans were identified in the green coffee that was analyzed in the present study, but this group of compounds was detected in the roasted coffee beans. Similar findings were reported by Fowble et al. (2019). In their study, the concentration of furans was 25-fold lower in green coffee than in roasted coffee [13]. In turn, furfuryl alcohol, which is a derivative of furans, was detected in this study. The differences in the determination results may be related to varietal differences or the coffee cultivation conditions [26]. Fowble et al. analyzed Coffea arabica green beans from Antigua, Colombia. Propane, 2-methyl-1-nitro accounted for the largest proportion (33.57%) among the volatile compounds in the analyzed green coffee. The other most abundant compounds were 4.5-difluoroacetate isomer (11.79%) and 2-furanmethanol, acetate (6.43%). In total, these three main compounds accounted for approximately 50% of VOCs in the green coffee.

Fluorine compounds are among the compounds that were found. The presence of fluorine compounds in coffee is also confirmed by the results of the studies that were conducted by by Wolska et al. [27]. Fluorine is naturally present in soil and water. Hence, its compounds may be present in coffee beans, as in the case of tea leaves [28]. The determination of the volatile, the semi-volatile, and the non-volatile contaminants, including fluorine volatile compounds, in coffee, tea, oil, and cocoa was also determined by Revel’skii et al. [29].

### 2.2. Analysis of Volatile Compounds in Roasted Coffee

**Figure 1:**
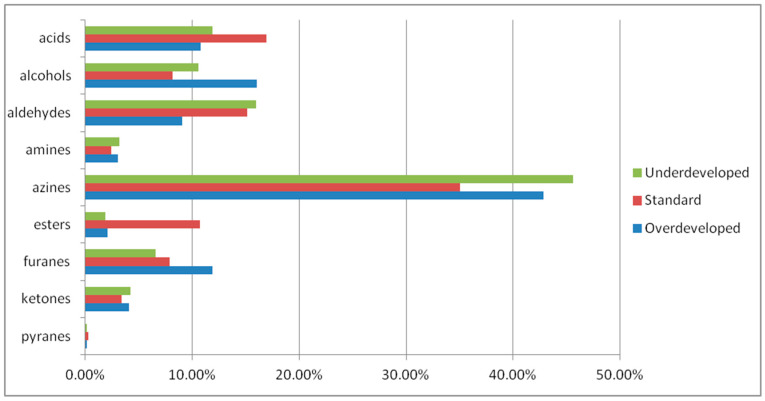
Percentage of the chemical classes of the volatile components of the 3 types of roasted coffee determined in the chromatographic analysis.

Table 2 shows 36 volatile compounds that were identified in the underdeveloped and the overdeveloped coffee. The volatile compounds were classified into relevant chemical groups, and their percentage is presented in Figure 1. Nine groups of compounds were distinguished, with a dominance of azines (underdeveloped: 45.65%, standard: 35.04%, and overdeveloped: 42.88%). A substantial amount was also determined in the case of aldehydes (underdeveloped: 15.96%, standard: 15.12%, and overdeveloped: 16.06%) and acids (underdeveloped 11.87%, standard 16.96%, and overdeveloped 10.75%). The analysis of the volatile fraction in the coffee beans that were roasted at the standard temperature and time identified 29 different compounds. In turn, the coffee that was roasted for a longer period of time (underdeveloped) exhibited the presence of 25 compounds, and 24 volatile compounds were identified in the coffee beans that were roasted more intensively for a shorter period of time (standard and overdeveloped).

Several compounds that are frequently present in roasted products were detected in the volatile fraction. These include furans, which are generated as by-products of sucrose degradation, pyrazine, which is derived from protein degradation, and pyridines, resulting from trigonelline degradation. These phenomena occur during the storage and the processing of coffee beans [30]. Heat-induced volatile compounds (furans, pyrazines, and pyridines) are more closely associated with the composition of roasted coffee aromas than esters, which are abundant in green coffee. One of the volatile substances was pyrimidine, 4,6-dimethyl-, whose content was 12.93% in the underdeveloped coffee and 10.11% and 11.31% in the standard and overdeveloped coffee, respectively. Another compound was 2-furanmethanol, which is otherwise known as furfuryl alcohol (underdeveloped coffee: 9.76%, standard coffee: 8.17%, and overdeveloped coffee 13.69%). This furan is a product of the Maillard reaction [31]. Considerable amounts of 2-furancarboxaldehyde, 5-methyl-, which is known as 5-methylfurfural, were detected as well. This product of the Maillard reaction gives food products the flavor of almonds, burnt sugar, and caramel. It represents the group of furans and aldehydes [32]. Its content was 7.12% in the underdeveloped coffee beans, 5.92% in the standard coffee beans, and 6.28% in the overdeveloped beans. Particular attention should also be paid to pyridine (underdeveloped: 9.89%, standard: 6.33%, and overdeveloped: 11.03%), which gives coffee its characteristic aroma. The presence of 2-furanmethanol acetate, which is otherwise known as furfuryl acetate (underdeveloped: 10.42%, standard: 6.54%, and overdeveloped: 10.75%) was detected as well. It gives the products a sweet and fruity aroma. Additionally, 2-acetolnyl-3-cyano-2,3-dimethylcyclobutane-1-carboxylic acid was found to be present in a substantial amount (9.91%) in the standard roasted coffee only. A compound that was identified during the analysis (pregnane-3,11,20,21-tetrol, cyclic 20,21-(butyl boronate), (3α,5β,11β,20R)) is a compound that is commonly found in plants. The same compound has also been identified by, among others, Ahmed et al., 2022 [33]; Gopu et al., 2021 [34]; and Aroosa et al., 2019 [35]. The term “volatile organic compounds” (VOCs) refers to the organic compounds that are present in the atmosphere as gases but can also be liquids or solids under normal conditions of temperature and pressure. The compound was not detected in the green coffee beans; however, thermal treatment caused its appearance in a volatile form and, therefore, it was adsorbed on the fiber, and was detected in the chromatographic analysis.

### 2.3. Statistical Analysis

**Figure 2:**
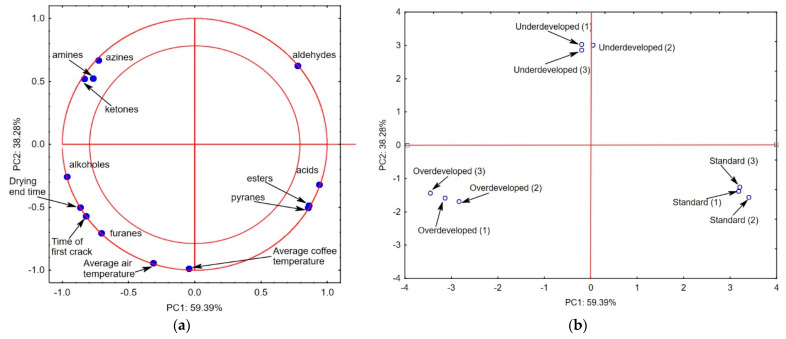
Projection of variables: volatile compounds characterizing the types of roasting, temperature of air, temperature of beans, and time to the first crack on the PC1 and PC2 scores plot—(**a**); projection of cases characterizing the type of roasting on the PC1 and PC2 loadings plot—(**b**).

Figure 2a shows the projection of the variables on planes PC1 (59.39%) and PC2 (38.28%), which describe the dependencies at 97.67%. Figure 2a shows a positive correlation between the time to the first crack, the average air temperature, the average coffee temperature, and the drying time and the percentage amounts of alcohols and furans. The correlation between these coffee roasting parameters and the content of aldehydes was negative.

The chemical compounds that are located in the area that is delineated by the two circles have a strong effect on the possibility of the indication of the coffee roasting method [5,15]. Amines, azines, ketones, and aldehydes are characteristic for the “underdeveloped” roast, whereas alcohols, furans, the drying time, the time to the first crack, the average air temperature, and the average coffee temperature describe the “overdeveloped” roast type. The “standard” roast mode is described by the content of acids, esters, and pyranes.

The chemical compounds and the roasting parameters that are located on the negative side of the main component PC2 distinguish the overdeveloped and the standard coffee from the underdeveloped variant. The first main component PC1 differentiates the standard and overdeveloped samples significantly. This actually suggests that the consumer may not perceive the flavors of the overdeveloped, the underdeveloped, and the standard roast coffee infusions as similar [36,37].

**Figure 3:**
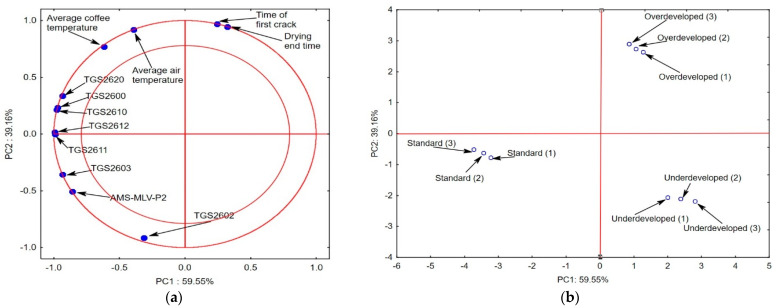
Projection of variables: responses of electronic nose sensors to different kinds of coffee roasting style, temperature of air, temperature of beans, and time to the first crack on the PC1 and PC2 scores plot—(**a**); projection of cases characterizing the type of roasting on the PC1 and PC2 loadings plot—(**b**).

Figure 3a shows the projection of variables (maximum electronic nose responses) on the PC1 (59.55%) and PC2 (39.16%) planes, which describe the dependencies at 98.71%,which is a very high level. The PC3 component represents 1.29%. The analysis revealed a positive correlation between the response of the electronic nose sensors TGS2620, TGS2600, and TGS2610, as well as TGS2612 and TGS2611, and the key roasting process parameters, i.e., the air and the coffee bean temperature. It also showed a negative correlation between the time to the first crack and the drying time and the TGS2603, AMS-MLV-P2, and TGS2602 sensor responses.

In this case, the responses of the electronic nose, which reacts to the intensity of the interactions of the volatile substances (odor level) [22], that are located in the area that is delineated by the two circles have a strong impact on the possibility of the indication of the roasting mode, especially in the case of the “standard” roast. The projection of the cases on the PC1 and PC2 planes (Figure 3b) shows that the two main components distinguish between the roasting styles; hence, the Agrinose is a suitable tool for the rapid identification of defective “overdeveloped” and “underdeveloped” roasting processes versus the correct “standard” roast. Our previous studies demonstrated the suitability of the e-nose for the identification of the regions of coffee origin and the content of pyridine in coffee [5,22].

## 3. Materials and Methods

### 3.1. Materials

The “Typica” variety of Arabica coffee beans was used in this study (See Supplementary Materials). It is cultivated in many regions of the world, but the coffee beans from Central America are of the highest quality. It is believed that the best “Typica” coffee beans are obtained from plantations located at altitudes exceeding 1600 m a.s.l. It has been observed that Central American coffee varieties that are grown in mountainous regions close to the Caribbean (Huehuetenango and Coban) or the Pacific (San Marcos) have a fruitier flavor and higher acidity. The “Typica” variety grown in Guatemala is well adapted to colder conditions and has moderate nutritional requirements, but is particularly sensitive to coffee leaf rust, coffee cherry disease, and pests. The first harvest is carried out from December to April in the fourth cultivation year. The harvested fruits are treated with the wet method to enhance their acidity and then they are dried in the sun. The SCA (Specialty Coffee Association) coffee roast level should be moderately light. The beans that are treated in this way have a floral-citrus, chocolate, or slightly nutty aroma, as well as pleasant and delicate acidity [38]. The content of the chemical and aromatic compounds contained in the green beans of this coffee variety was determined before the start of the roasting process. 

### 3.2. Roasting Procedure

**Figure 4:**
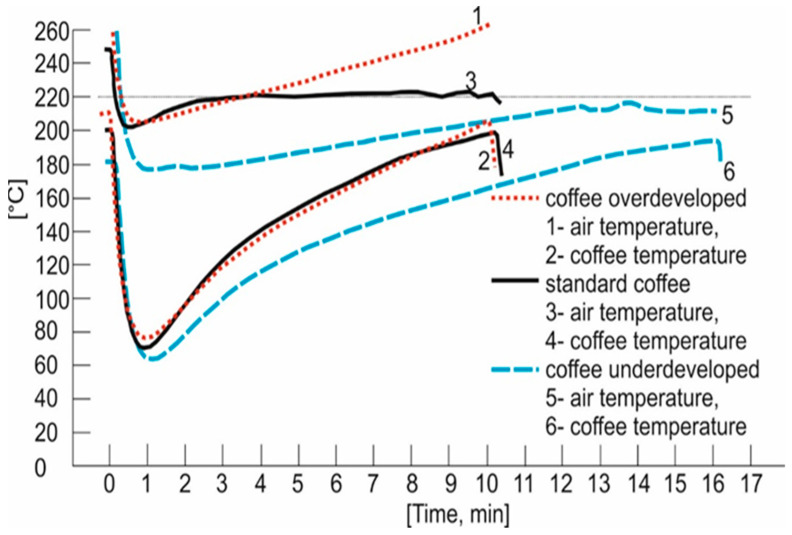
Temperature of coffee beans and air as a function of roasting time.

The coffee beans were roasted in a Rovigo Caffee roaster (Lublin, Poland). The beans were roasted in a Coffed SR 5 roaster equipped with a double-walled drum, as well as coffee and exhaust temperature sensors (Coffee Roasters, Piła, Poland). It offered the possibility of controlling the drum rotation speed and the combustion fan speed, air flow, and the burner power. Hence, it was possible to plot three curves as a function of time, namely the temperature in the roaster, the temperature of the coffee beans during roasting, and the increase in the temperature of the beans referred to as ROR (rate of rise). To achieve optimal and reproducible roasting conditions in the Coffed SR 5 roaster, each batch of beans represented a full load (5 kg). The coffee beans were roasted in three repetitions in for the same conditions. The following three modes of roasting the beans were used: roasting at an initial temperature of 240 °C to produce an overdevelopment defect, standard roasting at an initial temperature of 220 °C to obtain medium light beans, and roasting at an initial temperature of 210 °C to achieve an underdevelopment defect [39]. The process conditions for each type of roasting technique were recorded, and the temperature profiles of the air and coffee beans as a function of time are shown in Figure 4.

At the start of the roasting process, cold coffee beans enter the roaster that is set at a very high temperature of over 240 °C (curves 1, 3, and 5). Curves 2, 4, and 6, represent the temperature of a hot probe inside the roaster, which absorbs the thermal energy of hot air. The probe inside the roaster indicates 180 °C for the procedure of the underdeveloped roast, 200 °C for the standard roast procedure, and 240 °C for the procedure of the overdeveloped roast. At this stage, the cold beans absorb thermal energy, and the hot probe inside the roaster cools. This is represented by an initial drop on the curve, which lasts until the beans and the probe reach the same temperature. It is visible for the underdeveloped defect on curve 2 after 0.5 min, for the standard roasting on curve 4 after 1 min, and for the overdeveloped defect after 1.5 min. Afterwards, the beans and the probe reach the same temperature, and they will then rise in sync with each other. Once it is reached, this equilibrium is known as the “turning point”.

### 3.3. Electronic Nose

An Agrinose device, which was designed and constructed at the Institute of Agrophysics of the Polish Academy of Sciences in Lublin, was used to determine the volatile compound profile [5,40,41]. It has a matrix of eight different MOS gas sensors (TGS2600—general air contaminants, hydrogen, and carbon monoxide; TGS2602—ammonia, hydrogen sulfide, high sensitivity to VOC, and odorous gases; TGS2603—odors generated from spoiled foods; TGS2610—LP gas and butane; TGS2611—natural gas and methane; TGS2612—methane, propane, and butane; TGS2620—solvent vapors, volatile vapors and alcohol; AS–MLV-P2—CO, butane, methane, ethanol and hydrogen, which are specifically designed for volatile organic compounds). Based on the sensor response, three parameters characterizing the individual VOCs can be defined. These include the following: maximum sensor response ΔR/R_max_, response time t_IM_, which is measured from the start of the analysis to the achievement of the maximum sensor response, and cleaning time t_CL_, i.e., the time of removal of the odor from the sensor, which is measured from the end of the analysis to half of the ΔR/R_max_ value. The established parameters depended on the type of the volatile substances and the intensity of the emission of compounds contained in the odor profile [42,43].

### 3.4. GC–MSAnalysis

A Trace GC Ultra gas chromatograph (ThermoFisher Scientific, Waltham, MA, USA) coupled with an ITQ 1100 mass spectrometer (ThermoFisher Scientific, Waltham, MA, USA) was used to carry out the GC–MS analysis in accordance with a procedure described in other studies [5,22]. The SPME 50/30 µm Divinylbenzene/Carboxen/Polydimethylsiloxane (DVB/CAR/PDMS), Stableflex (2 cm) 24 Ga (Sigma Aldrich, Poznań, Poland) fiber was used for the chromatographic analyses. The fiber with the adsorbent was placed for 30 min in the measuring chamber, which contained VOC-emitting coffee beans (250 g) at the same temperature (22 °C), which was monitored during all measurements. Next, the fiber was transferred to the GC injector for 5 min to desorb the VOCs. A Zebron ZB-5Msplus Capillary GC 30 m × 0.25 mm × 0.25 μm capillary column was used in the analyses. The injection temperature was 60 °C for 5 min. Then, it was increased from 60 to 250 °C at a rate of 5 °C/min and from 250 °C to 270 °C at a rate of 10 °C/min. The final temperature was maintained for 5 min. The helium flow rate was kept constant at 2.2 mL/min. The temperature of the ion source transfer line was 280 °C. The electron ionization (EI+) mode with electron energy of 70 eV was applied. The mass spectrometer acquired data in the full scan mode (scan ranges: 35–390). Each variant of the experiment was performed in three repetitions. For the identification of the compounds, the Wiley 138 library was used for the highest quality of matching. The procedure was also described in other publications by the authors of the present study [22].

### 3.5. Statistical Analysis

Statistica software (version 12.0, StatSoft Inc., Tulsa, OK, USA) was used for the statistical analyses. Principal component analysis (PCA), analysis of variance, and the determination of correlations were performed at a significance level of α = 0.05. The principal component analysis was employed to determine the relationships between the maximum responses of the chemically sensitive sensors and the volatile compounds emitted from the coffee varieties in the two roasting methods [22,44]. The PCA data matrix for the statistical analysis of the results of the chromatographic tests had 13 columns (names of the volatile compounds) and 9 rows (type of coffee and type of roast). In turn, the PCA data matrix for the statistical analysis of the results provided by the electronic nose had 12 columns (type of sensors) and 9 rows (max responses—ΔR/Rmax). The input matrix was scaled automatically. The optimal number of principal components obtained in the analysis was determined based on the Cattel criterion.

## 4. Conclusions

The roast defects that are produced in processes that are carried out with differing parameters, i.e., the initial air temperature and the time to the first crack, were difficult to identify with the methods that are used in roasteries. The color of the underdeveloped beans did not differ from that achieved in the typical light smoking styles (light, light cinnamon), and the color of the overdeveloped beans did not differ from the dark roasted beans (Vienna or French roasting). The use of the Agrinose and the chemometric methods facilitated the identification of the volatile compound profile and the intensity of the aroma of the roast-defect coffee. The analysis of the volatile compounds in the roasted coffee beans helped to identify the compounds that distinguish the roast defects. Properly roasted coffee is characterized by a substantially higher percentage of esters and acids than that in overdeveloped and underdeveloped coffee. Overdeveloped coffees have a considerably higher amount of alcohols (15%) and furans (12%). In turn, underdeveloped coffees have the highest content of aldehydes (16%) and azines (46%). The statistical analysis of the Agrinose sensor responses facilitated the identification of the defects. The projection on the PC1 plane clearly differentiated between the overdevelopment and underdevelopment defects, and the projection on the PC2 plane discriminated between the properly roasted coffee and both of the roast defects.

## Figures and Tables

**Table 1:** Volatile compounds determined in the chromatographic analysis. %—percentage share of the compounds in the green coffee with standard deviations.

No.	R_time_	Name of Compound	Chemical Formula	Chemical Class	Percentage
1	1.87	4,5-difluoroctane isomer	C8H10F2	hydrocarbons	11.79 ± 0.39
2	3.09	8,11,14-eicosatrienoic acid, methyl ester	C21H36O2	esters	5.71 ± 0.30
3	4.05	2-furanmethanol	C5H6O2	alcohols	6.07 ± 0.14
4	5.82	2-methyl-3-(2-methylpropyl) pyrazine	C9H14N2	azines	4.29 ± 0.27
5	6.47	1,3,7-octatriene, 3,7-dimethyl-, E-	C10H16	terpenes	5.00 ± 0.13
6	7.44	5-amino-1-benzoyl-1H-pyrazole-3,4-dicarbonitrile	C12H7N5O	amines	4.64 ± 0.19
7	8.35	Propane, 2-methyl-1-nitro-	C4H9NO2	esters	33.57 ± 0.83
8	8.47	2-furanmethanol, acetate	C7H8O3	alcohols	6.43 ± 0.30
9	9.61	Oxiranecarboxamide, 2-ethyl-3-propyl-	C8H15NO2	others	1.79 ± 0.09
10	9.70	6,6-dimethyl-2-methylene-bicyclo [3.1.1] heptane	C10H16	terpenes	5.36 ± 0.15
11	9.86	2-cyclopropyl-2-methylspiro [2.2]pentane-1-carboxylic acid	C10H14O2	acids	3.93 ± 0.10
12	15.22	4-hydroxy-4-methyl-hex-5-enoic acid tert-butyl ester	C11H20O3	esters	2.5 ± 0.10
13	15.42	9,12,15-octadecatrienoic acid, methyl ester	C19H32O2	esters	1.07 ± 0.06
14	18.15	16-methylene-1,20-dioxopregn-4-en-17-yl acetate	C24H32O4	esters	1.07 ± 0.07
15	21.06	1,4-methanoazulene, decahydro-4,8,8-trimethyl-9-methylene-, [1S-(1α,3Aβ,4α,8Aβ)]-	C15H24	terpenes	1.07 ± 0.02
16	23.37	Ethanone, 1-(5,6,7,8-tetrahydro-2,8,8-trimethyl-4H-cyclohepta[B]furan-5-yl)-	C14H20O2	ketones	1.43 ± 0.03
17	31.28	Alanine, 3-(benzyloxy)-, L-	C10H13NO3	acids	1.07 ± 0.01
18	32.19	Hexadecadienoic acid, methyl ester	C17H30O2	esters	3.21 ± 0.02

**Table 2:** Volatile compounds determined in the chromatographic analysis. %—percentage share of the compound with standard deviations in the tested sample immediately after roasting of underdeveloped, standard, and overdeveloped conditions.

No.	R_time_	Name of Compound	Chemical Formula	Chemical Class	Underdeveloped	Standard	Overdeveloped
1	1.29	2-acetonyl-3-cyano-2,3-dimethylcyclobutane-1-carboxylic acid	C11H15NO3	acids	n.d.	9.91 ± 0.24	n.d.
2	1.61	Furan, 2-methyl-	C5H6O	furanes	5.67 ± 0.27	4.19 ± 0.22	6.7 ± 0.40
3	1.85	Butanal, 2-methyl-	C5H10O	aldehydes	2.77 ± 0.05	2.66 ± 0.11	2.37 ± 0.22
4	2.01	(2E)-2-(hydroxyimino)etyl acetate	C4H7NO3	esters	1.45 ± 0.10	n.d.	n.d.
5	2.42	Pyridine	C5H5N	azines	9.89 ± 0.35	6.33 ± 0.14	11.03 ± 0.69
6	3.14	Pregnane-3,11,20,21-tetrol, cyclic 20,21-(butyl boronate), (3α,5β,11β,20R)-	C25H43BO4	others	0.79 ± 0.03	n.d.	n.d.
7	3.17	2-butanone	C4H8O	ketones	3.69 ± 0.30	2.76 ± 0.08	2.51 ± 0.13
8	3.20	2-thiopheneethanol, 5-(4,5-dihydro-4,4-dimethyl-2-oxazolyl)-	C11H15NO2S	alcohols	n.d.	n.d.	2.37 ± 0.13
9	3.48	Pyrimidine, 2-methyl-	C5H6N2	azines	8.58 ± 0.30	8.17 ± 0.28	8.94 ± 0.34
10	3.60	2-furancarboxaldehyde	C5H4O2	aldehydes	5.8 ± 0.40	6.23 ± 0.25	n.d.
11	4.01	2-furanmethanol	C5H6O2	alcohols	9.76 ± 0.05	8.17 ± 0.22	13.69 ± 0.51
12	4.31	1,5-dimethyl-2,3-dihydro-1H-pyrrole	C6H11N	furanes	n.d.	3.06 ± 0.10	5.17 ± 0.29
13	5.50	Ethyl 2,3-pentadienoate	C7H10O2	esters	1.58 ± 0.10	n.d.	1.82 ± 0.13
14	5.63	2-amino-4-methyl-2-pentennitrile	C6H10N2	amines	3.03 ± 0.1	2.35 ± 0.12	2.93 ± 0.10
15	5.75	Pyrimidine, 4,6-dimethyl-	C6H8N2	azines	12.93 ± 0.63	10.11 ± 0.56	11.31 ± 0,90
16	5.86	Pyridine-2-D, 6-ethyl-	C7H8DN	azines	4.09 ± 0.10	3.17 ± 0.20	3.49 ± 0.22
17	7.35	2-furancarboxaldehyde, 5-methyl-	C6H6O2	aldehydes	7.12 ± 0.29	5.92 ± 0.25	6.28 ± 0.25
18	8.45	2-furanmethanol, acetate	C7H8O3	alcohols	10.42 ± 0.56	6.54 ± 0.31	10.75 ± 0.53
19	8.60	2-pyridinecarbonitrile, 1,2,5,6-tetrahydro-1-methyl-	C7H10N2	azines	4.75 ± 0.23	3.37 ± 0.17	3.91 ± 0.10
20	8.79	Pyrazine, 2-ethyl-5-methyl-	C7H10N2	azines	3.3 ± 0.16	2.25 ± 0.15	2.65 ± 0.20
21	11.28	Pyrazine, 3-ethyl-2,5-dimethyl-	C8H12N2	azines	1.98 ± 0.06	1.33 ± 0.12	1.54 ± 0.03
22	11.42	Furan, 2,2′-methylenebis-	C9H8O2	furanes	0.92 ± 0.07	0.61 ± 0.08	n.d.
23	11.69	2-cyclopenten-1-one, 3-ethyl-2-hydroxy-	C7H10O2	ketones	n.d.	n.d.	0.84 ± 0.03
24	14.51	1,1-dimethyl-1,3-dihydroisobenzofuran-3-one	C10H10O2	ketones	0.53 ± 0.05	n.d.	0.7 ± 0.05
25	14.79	2-hydroxymethylene-6-isopropyl-3-methyl-cyclohexanone	C11H18O2	ketones	n.d.	0.41 ± 0.05	n.d.
26	18.34	3,5-heptadienal, 2-ethylidene-6-methyl-	C10H14O	aldehydes	0.26 ± 0.05	0.31 ± 0.02	0.42 ± 0.02
27	19.50	Ethyl (2E,4E,6E)-9-formyl-10-oxo-2,4,6,8-decatetraenoate	C13H14O4	esters	n.d.	n.d.	0.14 ± 0.01
28	22.48	1H-2-benzopyran, 3-(3,4-dimethoxyphenyl)-6,7-dimethoxy-1-methyl-	C20H22O5	pyranes	0.13 ± 0.02	0.31 ± 0.01	0.14 ± 0.01
29	26.08	2,7-diphenyl-1,6-dioxopyridazino[4,5-2′,3′]pyrrolo[4′,5′-D]pyridazine	C20H13N5O2	azines	0.13 ± 0.03	0.31 ± 0.02	n.d.
30	26.18	4H-1-benzopyran-4-one, 2-(3,4-dimethoxyphenyl)-3,5-dihydroxy-7-methoxy-	C18H16O7	ketones	n.d.	0.2 ± 0.01	n.d.
31	28.39	Cholan-24-oic acid, 3,7,12-trihydroxy-, (3α,5β,7α,12α)-	C24H40O5	acids	n.d.	0.51 ± 0.01	n.d.
32	28.44	Tridecanoic acid, 12-methyl-, methyl ester	C15H30O2	esters	n.d.	0.31 ± 0.01	n.d.
33	31.28	1-isothiocyanato-3-methyladamantane	C12H17NS	amines	0.13 ± 0.02	0.1 ± 0.01	0.14 ± 0.01
34	32.19	Methyl (7E)-7-hexadecenoate	C17H32O2	esters	0.26 ± 0.03	7.05 ± 0.28	0.14 ± 0.01
35	32.64	Hexadecanoic acid, methyl ester	C17H34O2	esters	n.d.	2.96 ± 0.14	n.d.
36	34.60	Heptadecanoic acid, methyl ester	C18H36O2	esters	n.d.	0.41 ± 0.03	n.d.

n.d.—not detected.

## Data Availability

Not applicable.

## Supplementary Materials

The following supporting information can be downloaded at: https://www.mdpi.com/article/10.3390/molecules27238530/s1.
