# Supplementary material for: How to Identify Roast Defects in Coffee Beans Based on the Volatile Compound Profile
Source: Molecules. 2022 Dec 3;27(23):8530. doi: 10.3390/molecules27238530 (PMC9737409; doi:10.3390/molecules27238530)
Supplement: Supplementary file 1 [file molecules-27-08530-s001.zip › molecules-1940455-supplementary.pdf]

Supplementary Materials. Additional information

Information about coffee: Guatemala SHB ep Huehuetenango

- Coffee Grade: SHB EP
- Grower: Hugo Chávez Mendez
- Farm Size: 0.5 hectares
- Variety: Bourbon,
- Region: San Pedro Necta, Huehuetenango, Western Guatemala
- Harvest: October – March 2020
- Altitude: 1524 - 1650 masl (metres above see level)
- Soil: Clay minerals
- Process: Fully washed and dried in the sun
- EP (European Preparation) means that the green coffee was sorted by hand to remove any defective beans and foreign material.
